# Analysis of the therapeutic effect of acitretin capsules combined with cooling blood and detoxifying formula on psoriasis hemorrhagic fever and its impact on the gut microbiota of patients

**DOI:** 10.1097/MD.0000000000041502

**Published:** 2025-03-21

**Authors:** Mengyun Zhou, Zhaoyi Li, Jia Liu, Wei-Feng Zha, Yunyun Shan, Tianhong Xu

**Affiliations:** a Department of Dermatology, Hangzhou Third People’s Hospital, Hangzhou, China.

**Keywords:** acitretin capsules, cooling blood and detoxifying formula, gut microbiota, hemorrhagic fever, psoriasis

## Abstract

This study evaluates the therapeutic effect of acitretin capsules combined with a cooling blood and detoxifying formula (CBDF) on psoriasis hemorrhagic fever and its impact on the gut microbiota (GM). Seventy patients diagnosed with psoriasis hemorrhagic fever between January 2022 and April 2023 were divided into 2 groups: an experimental group (EG) treated with acitretin capsules and the CBDF, and a control group treated with acitretin capsules alone. The study assessed immune inflammatory responses, symptom improvement, traditional Chinese medicine symptom scores, and changes in GM diversity and abundance before and after treatment. After 2 months, the EG showed significant reductions in interleukin-2 receptor levels (from 813.2 ± 201.5–402.3 ± 111.2 mg/L) and interleukin-6 (from 8.4 ± 3.1–3.8 ± 1.7 pg/mL). The quality of life index improved by 6.12 points in the EG, which was significantly better than the control group (*P* < .05). PASI scores decreased in both groups, with the EG showing greater improvement (PASI before treatment: 23.1 ± 4.3; after 1 month: 15.6 ± 3.8; after 2 months: 10.4 ± 3.3). The EG also showed a significant increase in GM diversity after treatment (*P* < .05). Acitretin capsules combined with the CBDF is effective in treating psoriasis hemorrhagic fever and positively affects the GM, offering a potential new treatment approach.

## 1. Introduction

Psoriasis is a skin disease with chronic inflammatory that has a long course and is prone to recurrence.^[1]^ Among them, psoriasis hemorrhagic fever (PHF) is a traditional Chinese medicine (TCM) syndrome differentiation and classification of this disease, characterized by symptoms such as skin flushing, rapid rash development, and severe itching.^[2]^ With the development of modern medicine, more and more treatment methods are being applied to the treatment of psoriasis, including Western medicine therapies such as acitretin capsules (AC) and TCM cooling blood and detoxifying formula (CBDF) therapy.^[3]^ As a commonly used Western medicine for treating psoriasis, the main component of AC, retinoic acid, can achieve the effect of treating psoriasis by regulating the proliferation and differentiation of epidermal cells.^[4]^ However, although the use of AC alone can alleviate the condition to a certain extent, long-term use may bring some side effects and is prone to recurrence after discontinuation of the medication.^[5]^ Therefore, it is particularly important to find a treatment method that can increase efficacy, reduce toxicity, and reduce the recurrence rate. The CBDF of TCM is mainly effective in clearing heat and detoxifying, cooling blood, and promoting blood circulation. It can regulate the body’s internal condition, improve micro-circulation, and thus achieve the goal of treating psoriasis. Moreover, TCM formulas usually have fewer side effects and are more suitable for long-term use.^[6,7]^ In addition, in recent years, the relationship between gut microbiota (GM) and psoriasis has gradually received attention.^[8]^ As the largest micro-ecological system in the human body, GM not only participates in physiological processes such as nutrient absorption and metabolism, but is also closely related to immune regulation and disease occurrence and development.^[9]^ Therefore, exploring the changes in GM after treatment in PHF patients may give a new way for treating psoriasis. Based on the above background, this study analyzes the therapeutic effect of AC combined with CBDF on PHF and explores the impact of this combination therapy (AC–CBDF) on patient GM, in order to provide a more scientific and effective treatment plan for psoriasis. The innovation of this study is mainly reflected in 2 aspects. One is to innovatively combine AC with CBDF to explore a new model of combining traditional Chinese and Western medicine (TC/W-M) for the treatment of psoriasis. The second is to conduct in-depth analysis of the changes in the patient’s GM, attempting to reveal the impact of integrated TC/W-M treatment on the GM.

## 2. Materials and methods

### 2.1. General information

This study was approved by the Ethics Committee of Hangzhou Third People’s Hospital (2023-0921-k019). This study selected 70 PHF patients admitted to hospitals between January 2022 and April 2023 as the study subjects for retrospective analysis. All selected patients met the diagnostic criteria for PHF and excluded other diseases that may cause skin symptoms. The admission criteria for selected patients are as follows.^[10,11]^ ① Confirmed as PHF by clinical and/or pathological examination; ② between the ages of 18 and 80; ③ has not received any other relevant treatment, or has stopped treatment for a period of time to ensure washout period; ④ patients who can cooperate to complete the entire research process. The exclusion criteria are as follows: ① there are other serious systemic diseases present; ② recently received relevant treatment; ③ allergy to any component in AC or CBDF; ④ pregnant or lactating women; ⑤ patients who cannot take medication or follow-up according to research requirements. General information such as age, gender, course of illness, and severity of the condition of all patients were recorded in detail and statistically analyzed to ensure comparability between groups of patients. This study was carried out with the approval of the hospital ethics committee and with the consent of all patients and their families.

### 2.2. Research method

This study according to different treatment methods divide psoriasis patients into an experimental group (EG) and a control group (CG). EG patients received AC–CBDF treatment, while CG only received AC treatment. The treatment cycle for CG and EG is 2 months. CG takes oral AC once a day in the morning and evening during the treatment cycle, 10 mg each time; in addition to taking 10 mg of AC in the morning and evening, EG also needs to decoct 1 dose of CBDF every day, and take the juice twice in the morning and evening. The CBDF used in this study mainly includes the following medicinal materials: raw land, raw gypsum, hyoglossoglossum, comgrass 20 g each, white grass 30 g, tengma 10 g, radix erythae, honeysuckle, forsythia, and isatis 15 g each. These medicinal herbs together constitute CBDF, aimed at cooling blood and detoxifying, especially suitable for symptoms such as excessive toxins, damage to blood vessels, blood overflowing when encountering heat, and hot toxins forcing blood to act recklessly. By comparing the various observation indicators of 2 groups of patients before, during, and after treatment, to analyze the specific therapeutic effect of AC–CBDF on PHF and its impact on patient GM.

### 2.3. Observation indicators

To analyze the therapeutic effect of AC–CBDF on PHF and its impact on patient GM, there are mainly 10 observation indicators in this study. The first is the immune inflammatory response (IMFR) indicator, which mainly includes the levels of interleukin-2 (IL-2) receptor, interleukin-6 (IL-6), and tumor necrosis factor-α (TNF-α). These indicators were measured in pretreatment (PT), 1 month after treatment (1 MAT) and 2 months after treatment (2 MAT) to evaluate the impact of different treatment regimens on patient IMFR. The second indicator is the recurrence rate, which evaluates the persistence of treatment effects by comparing the recurrence rates of different skin injury symptoms (erythema, infiltration, scales) between 2 groups of patients. The third one is the TCM symptom score, which scores patients based on TCM symptoms (such as itching, restlessness, thirst, yellow urine, constipation, etc) and compares the changes before and after treatment to reflect the improvement of TCM treatment on patient symptoms.^[12]^ The fourth and fifth indicators are the Skin Disease Quality of Life Index (SDLQI) and Itching Degree Score, which are used to evaluate the patient’s SDLQI and itching degree before and after treatment, reflecting the impact of treatment on the patient’s quality of life (QoL) and symptom relief.^[13]^ The sixth indicator is the PASI score, which is mainly used to quantify the severity of psoriasis. By comparing the PASI score before and after treatment, the treatment effect is measured. Table 1 shows the specific grading of PASI scores.^[14]^

**Table 1:** Psoriasis lesion area and severity rating table.

Mark	Area of lesion	Individual symptoms of skin lesions		
		Erythema	Infiltrate	Scale
0 points	No skin lesions	Erythema free	No infiltrating hypertrophy	Non-scale
1 point	Skin lesions less than or equal to 10%	Light (pink/light red)	Mild (lesions slightly higher than normal skin surface)	Mild (some skin lesions with scales, mainly fine scales)
2 points	Lesions range from 11% to 30%	Medium (dark pink)	Moderate (easily palpable lesions with rounded or sloping edges)	Moderate (most lesions are not completely covered with scales and are flaky)
3 points	Lesions range from 31% to 50%	Severe (red)	Severe (clear edges, obvious ridges)	Severe (almost all skin lesions are scaly, with thick layers of scales)
4 points	Lesions range from 51% to 70%	Very heavy (dark red)	Extremely severe (highly infiltrated, eminently elevated)	Extremely severe (all skin covered with scales)
5 points	Lesions range from 71% to 90%	–	–	–
6 points	Lesions range from 91% to 100%	–	–	–

The seventh is clinical efficacy, which is divided into significant, effective, and ineffective based on the improvement of the patient’s symptoms. The total effective rate is calculated to evaluate the overall effectiveness of the treatment. The eighth is the adverse reaction rate (ARR), which calculates the total ARR by recording the adverse reactions that happen when treating to evaluate the safety of the treatment. The ninth is GM diversity. By comparing the CHao, Shannon, and Simpson values of GM after treatment between 2 groups of patients, the impact of treatment on GM diversity is evaluated. The last indicator is the correlation between GM changes and efficacy. Through Spearman rank correlation analysis (SRCA), the correlation between the therapeutic effect of AC–CBDF and GM changes is explored.

### 2.4. Statistical methods

This study used SPSS software for statistical analysis. Quantitative data was expressed as mean ± standard deviation, and independent sample *t* test was taken for comparison between 2 groups. Count data was represented by a percentage or frequency, and chi square test was utilized for inter-group comparison. For non-normal distribution or rank data, Mann–Whitney *U* test was used. The correlation analysis used SRCA. The comparison of GM diversity was conducted using multivariate statistical analysis methods to observe inter-group differences. When comparing the abundance of GM types or operational classifications, methods such as Metastats were used for differential analysis.^[15]^ All statistical tests were conducted using a double-sided test. *P* < .05 referred to statistically significant differences.

## 3. Results

### 3.1. Comparison of basic information of research subjects

Table 2 provides general information on the study subjects of EG and CG. There was no obvious discrepancy in gender, average age, average disease duration, PASI score, IL-2 receptor, IL-6, IL-8, TNF-α, TCM syndrome score (TCM-SC), SDLQI, and itching degree score between the 2 groups (*P* > .05).

**Table 2:** Comparison of general data of research objects.

Item	EG	CG	t	*P*
Total (n)	35	35	–	.693
Male cases (n)	18	17	–	.665
Female cases (n)	17	18	–	.652
Average age (years)	49.2 ± 11.8	47.8 ± 12.4	0.484	.315
Average duration of disease (years)	5.8 ± 3.1	5.4 ± 3.3	0.523	.302
PASI score	23.1 ± 4.3	22.5 ± 3.9	0.611	.272
IL-2 receptor (mg/L)	813.2 ± 201.5	821.3 ± 199.6	0.169	.433
IL-6 (pg/mL)	8.4 ± 3.1	8.5 ± 3.0	0.137	.446
TNF-α (pg/mL)	8.8 ± 2.8	8.9 ± 2.7	0.152	.440
TCM-SC	7.7 ± 2.1	7.6 ± 1.9	0.208	.418
SDLQI	13.8 ± 1.8	13.6 ± 1.6	0.491	.312
Pruritus score	4.12 ± 2.05	4.23 ± 2.11	0.221	.413

CG = control group, EG = experimental group, IL-2 = interleukin-2, IL-6 = interleukin-6, SDLQI = Skin Disease Quality of Life Index, TCM-SC = traditional Chinese medicine syndrome score, TNF-α = tumor necrosis factor-α.

### 3.2. Changes in IMFR indicators

To evaluate the impact of AC–CBDF on IMFR in PHF patients, this study compared multiple IMFR indicators of EG and CG in PT, 1 MAT, and 2 MAT. The specific comparison results are shown in Table 3. After 1 month and 2 months of treatment, the various IMFR indicators of EG were significantly reduced compared to PT (*P* < .05), and the various indicators of EG in 1 MAT and 2 MAT were significantly better than CG (*P* < .05).

**Table 3:** Multiple IMFR indexes among patients in different time periods.

Time node		IL-2 receptor (mg/L)	IL-6 (pg/mL)	TNF-α (pg/mL)
Pretreatment	CG	821.3 ± 199.6	8.5 ± 3.0	8.9 ± 2.7
	EG	813.2 ± 201.5	8.4 ± 3.1	8.8 ± 2.8
	t	0.169	0.137	0.152
	*P*	.433	.446	.440
1 MAT	CG	785.3 ± 177.5	8.0 ± 2.7	8.3 ± 2.5
	EG	618.5 ± 153.3*	6.1 ± 2.2*	6.3 ± 2.3*
	t	4.208	3.227	7.447
	*P*	.000	.001	.000
2 MAT	CG	753.2 ± 159.4	7.4 ± 2.3	7.7 ± 2.2
	EG	402.3 ± 111.2*	3.8 ± 1.7*	4.4 ± 1.8*
	t	9.371	7.447	6.868
	*P*	.000	.000	.000

CG = control group, EG = experimental group, IL-2 = interleukin-2, IL-6 = interleukin-6, IMFR = immune inflammatory response, TNF-α = tumor necrosis factor-α.

* An obviously significant difference compared to pretreatment (*P* < .05).

### 3.3. Comparative analysis of patient recurrence rates

**Figure 1.**
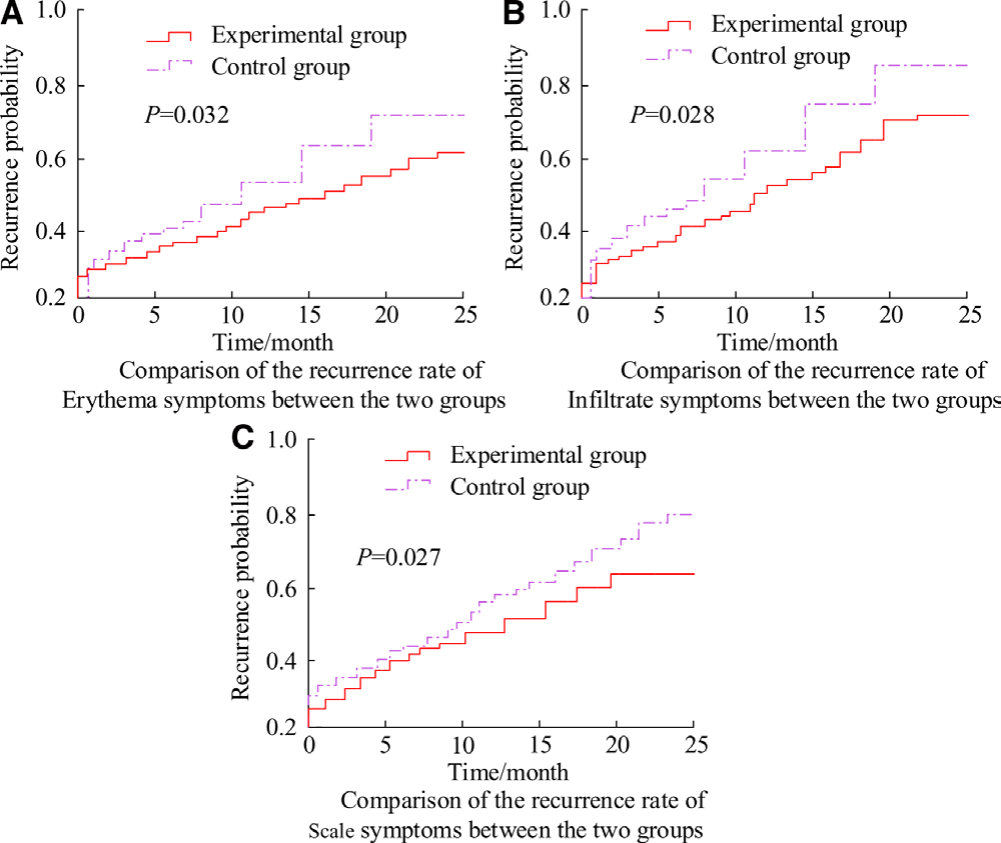
Comparison of recurrence rates of different SIS between the 2 groups. SIS = skin injury symptoms.

Figure 1 shows the comparison of recurrence rates between 2 groups of patients with different skin injury symptoms (SIS). In Figure 1A–C, there were obvious differences in the recurrence rates of erythema SIS (*P* = .032), invasive SIS (*P* = .028), and scale SIS (*P* = .027) between the 2 groups of patients.

### 3.4. Analysis of TCM syndrome scoring

To verify the impact of AC–CBDF on the TCM syndrome of PHF patients, the TCM-SC of patients B&A treatment were compared, and the comparison results are listed in Table 4. There were *P* < .05 in the TCM-SC and total scores of EG patients before and after treatment, while there was *P* > .05 in the scores of CG patients before and after treatment.

**Table 4:** Comparison of TCM syndrome score (TCM-SC) among cases before and after treatment.

Symptom	Group	Pretreatment	Posttreatment	t	*P*
Pruritus	EG	2.26 ± 0.65	0.99 ± 0.35	10.176	.000
	CG	2.23 ± 0.64	2.04 ± 0.48	1.405	.083
Be upset	EG	1.32 ± 0.68	0.61 ± 0.42	5.256	.000
	CG	1.33 ± 0.71	1.08 ± 0.66	1.537	.065
Thirstily	EG	1.55 ± 0.66	0.65 ± 0.48	6.524	.000
	CG	1.54 ± 0.68	1.21 ± 0.51	1.601	.057
Yellow urine	EG	1.35 ± 0.75	0.51 ± 0.47	5.615	.000
	CG	1.33 ± 0.77	1.05 ± 0.61	1.445	.077
Constipation	EG	1.17 ± 0.77	0.52 ± 0.46	4.287	.000
	CG	1.18 ± 0.78	0.98 ± 0.53	1.255	.107
Total points	EG	7.65 ± 2.07	3.58 ± 1.25	9.957	.000
	CG	7.61 ± 1.94	5.86 ± 1.71	2.539	.007

CG = control group, EG = experimental group, TCM = traditional Chinese medicine.

### 3.5. Analysis of SDLQI and itching severity score

**Figure 2.**
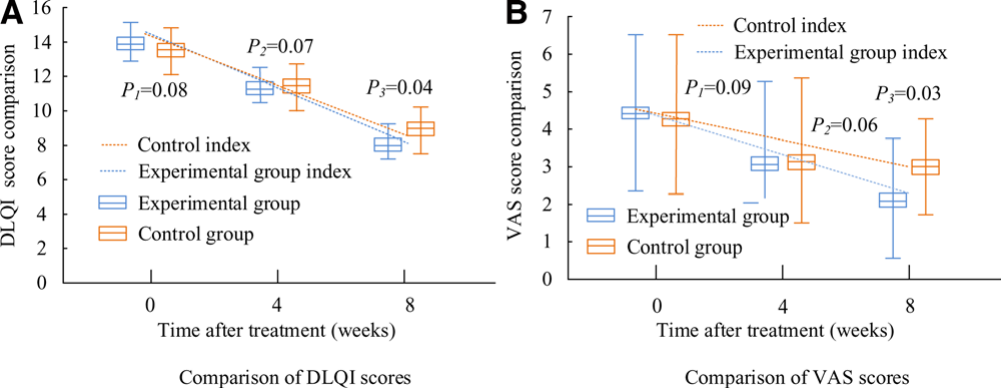
Comparison results of SDLQI and itch degree score before and after treatment between the 2 groups. SDLQI = Skin Disease Quality of Life Index.

Figure 2 shows the comparison of SDLQI and itching scores between 2 groups. In Figure 2A, there was *P* > .05 in SDLQI between the 2 groups of patients in PT and 1 MAT, but the difference was significant in 2 MAT (*P* = .04). In Figure 2B, there was *P* > .05 in the itching score among patients in PT and 1 MAT, but the difference was significant after 2 months of treatment (*P* = .03).

### 3.6. PASI score comparison

Table 5 shows the comparative PASI scores of patients before and after treatment. The PASI scores of EG patients in PT, 1 MAT, and 2 MAT were 23.1 ± 4.3, 15.6 ± 3.8, and 10.4 ± 3.3, respectively. The PASI scores of CG patients in 3 stages were 22.5 ± 3.9, 20.5 ± 3.6, and 18.2 ± 3.4. There was *P* < .05 between the 2 groups in 1 MAT and 2 MAT.

**Table 5:** Comparison of PASI scores between the 2 groups at different time points.

Time point	Group	PASI score	t	*P*
Pretreatment	EG	23.1 ± 4.3	0.611	.272
	CG	22.5 ± 3.9		
1 MAT	EG	15.6 ± 3.8*	5.538	.000
	CG	20.5 ± 3.6		
2 MAT	EG	10.4 ± 3.3*	9.739	.000
	CG	18.2 ± 3.4*		

CG = control group, EG = experimental group.

*Indicates statistically significant.

*Note*: * indicates a statistically significant difference compared to before treatment (*P* < .05).

### 3.7. Clinical efficacy comparison

After the end of the 2 month treatment cycle, this study comprehensively evaluated the clinical efficacy of EG and CG patients and recorded in detail the adverse reactions that occurred during the treatment process. Table 6 shows the specific results. The total effective rate of EG was greatly outperformed than CG (*P* < .05), while there was *P* > .05 in the total ARR.

**Table 6:** Comparison of clinical efficacy and adverse reactions between the 2 groups.

Item		EG (n = 35)	CG (n = 35)	*χ*²	*P*
Clinical effect	Remarkable	26	16	5.952	.015
	Effective	8	10	0.299	.584
	In vain	1	9	7.467	.006
	Total effective rate	94.3%	74.3%	6.158	.035
Adverse reaction	Pruritus	2	2	0.000	1.000
	Dry peeling	1	1	0.000	1.000
	Liver injury	0	1	1.014	.314
	Gastrointestinal reaction	1	1	0.000	1.000
	Hyperlipemia	1	2	0.348	.555
	Other	2	1	0.348	.555
	Total ARR	20.0%	22.9%	0.002	.960

ARR = adverse reaction rate, CG = control group, EG = experimental group.

### 3.8. Comparative analysis of GM diversity between 2 groups of patients

**Figure 3.**
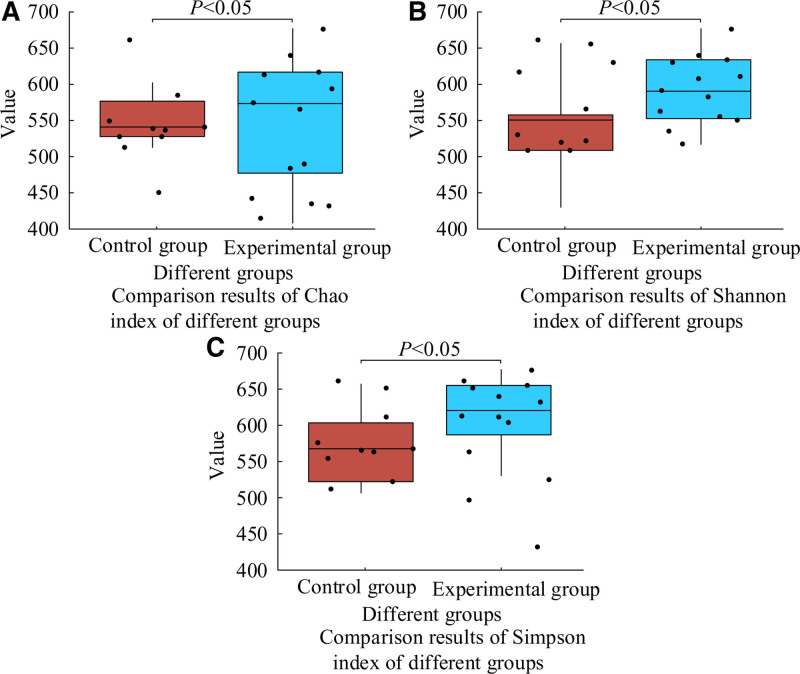
Diversity of GM in the 2 groups after treatment. GM = gut microbiota.

To explore the impact of AC–CBDF on the GM of PHF patients, this study compared the GM diversity of 2 groups of patients after 2 months of treatment, as shown in Figure 3. In Figure 3, there were *P* < .05 in the CHao, Shannon, and Simpson values of GM between the 2 groups of patients after treatment.

### 3.9. Correlation analysis between changes in GM and efficacy of AC–CBDF

**Figure 4.**
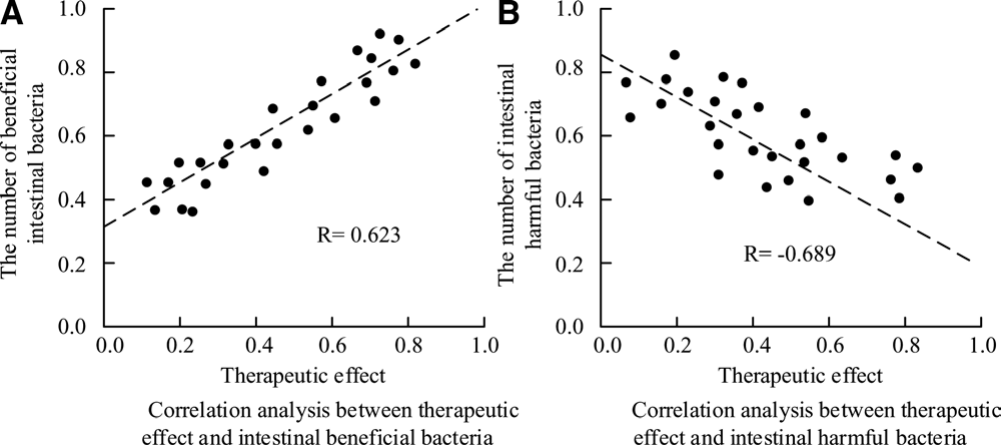
Correlation analysis of GM changes and the therapeutic effect of AC–CBDF. AC = acitretin capsules, CBDF = cooling blood and detoxifying formula, GM = gut microbiota.

The changes in GM in EG patients and the therapeutic effect of AC–CBDF were analyzed using SRCA, as shown in Figure 4. There was a significant correlation between the therapeutic effect of AC–CBDF and the changes in GM in PHF patients. Specifically, patients with effective treatment showed a significant increase in GM diversity, and the relative abundance of beneficial bacteria also increased, while the relative abundance of harmful bacteria decreased.

## 4. Discussion and conclusion

Psoriasis is a chronic inflammatory skin disease characterized by the appearance of red patches on the skin, covered with silver white scales. In TCM theory, psoriasis is often related to factors such as blood heat and blood stasis, so TCM often uses the method of cooling blood and detoxifying to treat it. However, traditional single TCM or Western medicine treatment methods often struggle to achieve ideal therapeutic effects. Therefore, it is particularly important to find an effective treatment method that combines TC/W-M.^[16,17]^ In recent years, with the continuous deepening of medical research, people have begun to pay attention to the association between GM and various diseases. As an important micro-ecological system in the human body, GM plays an important role in keeping human health and regulating immune function.^[18]^ An increasing number of studies suggest that the imbalance of GM may be closely related to the onset and development of inflammatory diseases such as psoriasis. In this context, the combination of TC/W-M treatment for AC–CBDF has emerged. As a retinoic acid class drug, AC can well regulate the differentiation and proliferation of skin cells, and alleviate skin inflammation. CBDF can clear heat and detoxify, cool blood and moisten dryness, and treat psoriasis from the perspective of TCM.^[19]^ The PHF treatment method combining AC and CBDF aims to comprehensively leverage the advantages of the 2 medical systems in order to achieve better therapeutic effects. At the same time, this study also analyzed the impact of this combined treatment method on the patient’s GM. By comparing and analyzing the changes in GM before and after treatment, it is hoped to further reveal the intrinsic relationship between GM and psoriasis, and provide new ideas for future treatment.

This study compared the various indicators of 2 groups of PHF patients B&A treatment to analyze the specific effect of AC combined with CBDF and its impact on patient GM. The comparison of IMFR indicators showed that after 2 months of treatment, the IL-2 receptor content in EG patients decreased from 813.2 ± 201.5 mg/L to 402.3 ± 111.2 mg/L, IL-6 decreased from 8.4 ± 3.1 pg/mL to 3.8 ± 1.7 pg/mL, and TNF-α decreased from 8.8 ± 2.8 to 4.4 ± 1.8. The changes in all IMFR indicators were statistically significant (*P* < .05). This result was similar to that obtained by Mallika et al in their study of PHF.^[20]^ The above results indicated that the combination of AC and CBDF could effectively alleviate the IMFR in PHF patients, which was expected to alleviate the condition and reduce skin lesions. This was closely related to the immune regulatory effect of AC and the anti-inflammatory and detoxifying effects of CBDF. In the comparison of recurrence rates, it was found that EG patients had significantly lower recurrence rates in SIS such as erythema, infiltration, and scales compared to CG. Komine team also obtained similar experimental conclusions in 2021.^[21]^ This indicated that AC–CBDF could not only alleviate current symptoms, but also to some extent prevented disease recurrence. The TCM-SC showed that the total TCM-SC of EG patients decreased from 7.65 ± 2.07 to 3.58 ± 1.25, while the score of CG patients decreased from 7.61 ± 1.94 to 5.86 ± 1.71, and the difference in data between the 2 groups was significant (*P* < .05). This result further confirmed the effectiveness of AC–CBDF in improving TCM symptoms in PHF patients.

According to the analysis of LQI and itching severity score, although there was no significant difference between the 2 groups of patients in PT and 1 MAT (*P* > .05). However, after 2 months of treatment, the LQI of EG patients decreased by 6.12 points compared to PT, and the itching score decreased by 2.41 points, both of which were significantly better than CG (*P* < .05). This result was consistent with the results obtained by Chularojanamontri team in their 2023 study on the clinical efficacy of psoriasis treatment methods.^[22]^ The above results indicated that with the deepening of treatment, AC–CBDF had an increasingly significant effect on improving the QoL and alleviating itching symptoms in patients.

The PASI score is an important indicator for measuring the severity of psoriasis. In this study, the PASI scores of patients with EG and CG in PT, 1 MAT, and 2 MAT were 23.1 ± 4.3, 15.6 ± 3.8, 10.4 ± 3.3, and 22.5 ± 3.9, 20.5 ± 3.6, and 18.2 ± 3.4, respectively. Yatsuzuka team also reached similar conclusions in their study of psoriasis treatment plans.^[23]^ The above results objectively reflected the significant therapeutic effect of AC–CBDF in improving the degree of skin lesions in PHF patients. From the overall evaluation of clinical efficacy, the total effective rate (94.3%) of EG patients was significantly higher than CG (74.3%), and there was *P* > .05 in total ARR. This result indicated that AC–CBDF not only had a definite therapeutic effect, but also had good safety. In addition, it was found that AC–CBDF had a positive impact on patient GM. As an important human microbiota, GM was closely related to the health status of its host. After treatment, the diversity of GM in EG patients significantly increased (*P* < .05), the relative abundance of beneficial bacteria grew, and the harmful bacteria lowed down. This result coincided with the research findings of Warren et al.^[24]^ This discovery not only revealed the potential connection between GM and the pathogenesis of psoriasis, but also provided new ideas for the future treatment of psoriasis by regulating GM. Further correlation analysis showed a significant correlation (*P* < .05) between the therapeutic effect of AC–CBDF and the changes in GM in PHF patients. Bhakthavatsalam et al and Miao also obtained similar conclusions in the study of the pathogenesis of psoriasis.^[25,26]^ This result suggested that the improvement of GM might be one of the important ways for this combination therapy to exert therapeutic effects. By regulating GM, it could help alleviate inflammatory reactions, improve immune function, and thus having a therapeutic effect on psoriasis.

Given the retrospective design of this study and the reliance on self-reported measures, recall bias and subjective evaluations may have influenced the findings. Additionally, the study’s sample size and the potential for missing data may limit the generalizability of the results. The absence of long-term follow-up data is another limitation, as it prevents an assessment of the lasting effects of the interventions. Future research should aim to address these limitations by incorporating larger, more diverse sample sizes, utilizing objective indicators such as biomarkers or imaging assessments to reduce bias, and conducting longitudinal studies to better understand the long-term outcomes of the interventions.

In summary, the combination therapy of AC and CBDF has shown significant effects in the PHF treatment. This combination of TC/W-M not only effectively reduces the patient’s immune inflammatory response, but also demonstrates outstanding performance in improving TCM symptoms and reducing recurrence rates. At the same time, this therapy has significantly improved the QoL of patients and alleviated itching symptoms. In addition, this combination therapy has a positive impact on the patient’s GM, increasing microbial diversity and regulating the balance between beneficial and harmful bacteria, providing a new perspective for the psoriasis treatment. The significant correlation between the improvement of GM and the therapeutic effect further reveals the vital role of GM in the pathogenesis and treatment of psoriasis. In the future, with the deepening of research, it is expected to bring safer and more effective treatment plans for psoriasis patients by regulating new treatment methods such as GM.

## Author contributions

**Conceptualization:** Mengyun Zhou.

**Data curation:** Mengyun Zhou.

**Formal analysis:** Mengyun Zhou.

**Investigation:** Mengyun Zhou.

**Methodology:** Mengyun Zhou.

**Writing – original draft:** Mengyun Zhou.

**Writing – review & editing:** Mengyun Zhou.

## References

[1] Choon SE Navarini AA Pinter A. Clinical course and characteristics of generalized pustular psoriasis. Am J Clin Dermatol. 2022;23:21–9. 35061227 10.1007/s40257-021-00654-z PMC8801409

[2] Zheng J Chen W Gao Y. Clinical analysis of generalized pustular psoriasis in Chinese patients: a retrospective study of 110 patients. J Dermatol. 2021;48:1336–42. 34018629 10.1111/1346-8138.15958 PMC8453703

[3] Yu Y Wang L. Study on the rule of traditional Chinese medicine in treating psoriasis with blood heat syndrome based on data mining. MEDS Chin Med. 2023;5:42–9.

[4] Qi L Liwen W. The understanding and treatment of common psoriasis in Chinese medicine. MEDS Chin Med. 2023;5:26–33.

[5] Lin YC Jeng YC Aala WJF. Transcriptomic responses of peripheral blood mononuclear cells to cyclosporin and etanercept in a female infant with juvenile generalized pustular psoriasis. Exp Dermatol. 2023;32:1299–305. 37194367 10.1111/exd.14835

[6] Trai NN Van Em D Van BT. Correlation of IL36RN and CARD14 mutations with clinical manifestations and laboratory findings in patients with generalised pustular psoriasis. Indian J Dermatol Venereol Leprol. 2023;89:378–84. 36331855 10.25259/IJDVL_1054_2021

[7] Du Y Yan Q Chen M Dong Z Wang F. Efficacy of adalimumab in pediatric generalized pustular psoriasis: case series and literature review. J Dermatolog Treat. 2022;33:2862–8. 35695300 10.1080/09546634.2022.2089327

[8] Rahman M Almalki WH Panda SK. Therapeutic application of microsponges-based drug delivery systems. Curr Pharm Des. 2022;28:595–608. 35040411 10.2174/1381612828666220118121536

[9] Chinthaginjala H Bogavalli V Hindustan AA. Nanostructured lipid carriers: a potential era of drug delivery systems. Ind J Pharm Edu Res. 2024;58:21–33.

[10] Amra K Momin M Desai N Khan F. Therapeutic benefits of natural oils along with permeation enhancing activity. Int J Dermatol. 2022;61:484–507. 34310695 10.1111/ijd.15733

[11] Verma N Rama A Jha A. Nanosponges: advancement in nanotherapeutics. Res J Pharm Technol. 2022;15:4253–60.

[12] Rana N Gupta P Singh H Nagarajan K. Role of bioactive compounds, novel drug delivery systems, and polyherbal formulations in the management of rheumatoid arthritis. Comb Chem High Throughput Screen. 2024;27:353–85. 37711009 10.2174/1386207326666230914103714

[13] Rivera-Diaz R Epelde F Heras-Hitos JA. Generalized pustular psoriasis: practical recommendations for Spanish primary care and emergency physicians. Postgrad Med. 2023;135:766–74. 38019177 10.1080/00325481.2023.2285730

[14] Songül I DEMİREL GZ AKIN SB Börü T. Multiple sclerosis in a patient with familial Mediterranean fever and psoriasis: a case report and review of the literature. Arel Üniversitesi Sağlik Bilimleri Dergisi. 2023;7:33–7.

[15] Reich K Augustin M Gerdes S. Generalized pustular psoriasis: overview of the status quo and results of a panel discussion. JDDG. 2022;20:753–71. 10.1111/ddg.14764 35674482

[16] Saeki H Mabuchi T Asahina A. English version of Japanese guidance for use of biologics for psoriasis (the 2022 version). J Dermatol. 2023;50:41–68. 10.1111/1346-8138.16691 36582113

[17] Burden AD. Spesolimab, an interleukin-36 receptor monoclonal antibody, for the treatment of generalized pustular psoriasis. Exp Rev Clin Immunol. 2023;19:473–81. 10.1080/1744666X.2023.2195165 36960829

[18] Singh A Choudhary R Chhabra N Ganguly S Rathore V. Severe pancytopenia following single dose methotrexate in psoriasis: a rare and potentially lethal manifestation. Curr Drug Saf. 2021;16:110–3. 33106147 10.2174/1574886315666201026125149

[19] Li XN Peng B Geng SM. Characterization of generalized pustular psoriasis in Northwest China: a single-center retrospective study. Int J Dermatol Venereol. 2022;5:191–8.

[20] Mallika CSL. Case study and patient counseling for elderly patient on psoriasis vulgaris. Int J Pharm Res Life Sci. 2022;10:25–9.

[21] Komine M Morita A. Generalized pustular psoriasis: current management status and unmet medical needs in Japan. Exp Rev Clin Immunol. 2021;17:1015–27. 10.1080/1744666X.2021.1961580 34402355

[22] Chularojanamontri L Rattanakorn K Julanon N Chuamanochan M Griffiths CE. Acrodermatitis continua of Hallopeau and generalised pustular psoriasis: should they be the same or different entities? Exp Dermatol. 2023;32:1235–45. 37057764 10.1111/exd.14805

[23] Yatsuzuka K Murakami M Kuroo Y. Flare‐up of generalized pustular psoriasis combined with systemic capillary leak syndrome after coronavirus disease 2019 mRNA vaccination. J Dermatol. 2022;49:454–8. 34862669 10.1111/1346-8138.16271

[24] Warren RB Reich A Kaszuba A. Imsidolimab, an anti-interleukin-36 receptor monoclonal antibody, for the treatment of generalized pustular psoriasis: results from the phase II GALLOP trial. Br J Dermatol. 2023;189:161–9. 37120722 10.1093/bjd/ljad083

[25] Bhakthavatsalam A Shivanna R. Ulcerations in chronic plaque psoriasis: a diagnostic clue for acute methotrexate toxicity. Clin Dermatol Rev. 2023;7:89–91.

[26] Miao C Chen Y Wang Z Xiang X Liu Y Xu Z. Real‐world data on the use of secukinumab and acitretin in pediatric generalized pustular psoriasis. J Dermatol. 2023;50:258–61. 35983654 10.1111/1346-8138.16551

